# Ascorbic Acid Sensor Based on CdS QDs@PDA Fluorescence Resonance Energy Transfer

**DOI:** 10.3390/molecules27072097

**Published:** 2022-03-24

**Authors:** Pu Li, Xiaoxiao Chen, Gaojun Wu, Zhe Wang, Chaobiao Huang

**Affiliations:** 1 College of Chemistry and Life Science, Zhejiang Normal University, Jinhua 321004, China; 202020200588@zjnu.edu.cn (P.L.); 26947255692@zjnu.edu.cn (X.C.); 2 Xingzhi College, Zhejiang Normal University, Jinhua 321004, China; 202120200767@zjnu.edu.cn (G.W.); L15839984772@163.com (Z.W.)

**Keywords:** CdS quantum dots, PDA, fluorescence resonance energy transfer, ascorbic acid

## Abstract

An ascorbic acid (AA) sensor was constructed based on the fluorescence resonance energy transfer (FRET) between CdS quantum dots (CdS QDs) and polydopamine (PDA) to detect trace AA sensitively. FRET occurred due to the broad absorption spectrum of PDA completely overlapped with the narrow emission spectrum of CdS QDs. The fluorescence of CdS QDs was quenched and in the “off” state. When AA was present, the conversion of DA to PDA was hindered and the FRET disappeared, resulting in the fluorescence of CdS QDs in an “on” state. Importantly, the degree of fluorescence recovery of CdS QDs displayed a desirable linear correlation with the concentration of AA in the range of 5.0–100.0 μmol/L, the linear equation is $y=0.0119c_{AA}+0.3113$, and the detection limit is 1.16 μmol/L (S/N = 3, *n* = 9). There was almost no interference with common amino acid, glucose and biological sulfhydryl small molecules to AA. Trace amount of AA in vitamin C tablets were determined and satisfactory results were obtained; the recoveries were observed to be 98.01–100.7%.

## 1. Introduction

The ascorbic acid (AA), a highly reductive polyhydroxy compound, is broadly used in the food industry as a source of vitamin C (Vc) or as an antioxidant, which acts as a key co-enzyme involved in a variety of indispensable biosynthetic processes such as amino acid metabolism, immune function, maintenance, non-heme iron absorption and so on in the human body [1,2,3]. Clinical trials have exhibited that AA supplementation produces antidepressant effects and improves mood [4]. However, as an essential nutrient that cannot be synthesized directly by the body, AA needs to be consumed from external foods and beverages. AA levels are associated with many diseases. Insufficient AA intake can lead to scurvy, while excessive AA intake can lead to kidney stones and stomach cramps [5,6]. Recent research has demonstrated the role of AA in stress-related disorders, inhibiting the development of cancer, and preventing and treating infection against COVID-19, making it urgent to analyze AA concentrations in food and clinical fields, especially for the performance measure of the techniques such as selectivity, sensitivity, and limit of detection in their utilization [4,7,8,9,10].

A range of analytical tools for the detection of AA have been proposed, including colorimetry [11,12,13], UV-vis spectrophotometry [14], electrochemical analysis [15,16,17,18,19], capillary electrophoresis [20,21], fluorescence analysis [22,23,24] and chromatography [25,26]. However, they are limited by the complexity of operations, low sensitivity or selectivity, expensive equipment, and time constraints. Electrochemical assay requires complex electrode modification procedures. Among them, the fluorescence method draws more attention owing to its high sensitivity, rapid response and operational simplicity, which makes for more potential in AA detection. Thus, designing a simple and efficient sensor for AA detection is essential. A considerable number of assays based on the FRET have been presented. For example, Wu et al. designed carbon quantum dots as the FRET organophosphate pesticides determination [27]. Tian et al. presented a FRET aptasensor between nanoceria and graphene quantum dots for the determination of ochratoxin A [28].

Dopamine (DA) existing in the form of hydrochloride is a catecholamine neurotransmitter regulating many biological processes in the brain. Under alkaline conditions, it can be oxidized to dopamine quinone and self-polymerizes in aqueous solution to form a series of oligomers with different molecular weights, while dopamine, dopamine quinone and their oligomers self-assemble in solution to form assemblies of different structures through various non-covalent bonds, collectively referred to as polydopamine (PDA) [29]. PDA has been proven as a fluorescence-quenching agent with good dispersibility, stability in aqueous solution, a wide absorption spectrum and high extinction coefficient [30,31,32,33,34]. It is reported that the fluorescence of FITC-labeled ssDNA can be quenched by PDA to the fluorescence “off” state through FRET. However, the spontaneous oxidative polymerization reaction from DA to PDA would be blocked, leading to the fluorescence “on” state when there are various antioxidants [35,36].

As semiconductor nanoparticles, QDs have become a heated topic of research owing to their high quantum yield, broad absorption spectrum, tunable emission and high optical stability, as well as their good dispersion in aqueous solution and good biocompatibility [37,38,39]. Studies have shown that QDs have been applied to life science, semiconductor devices and other fields. 

**Figure 1:**
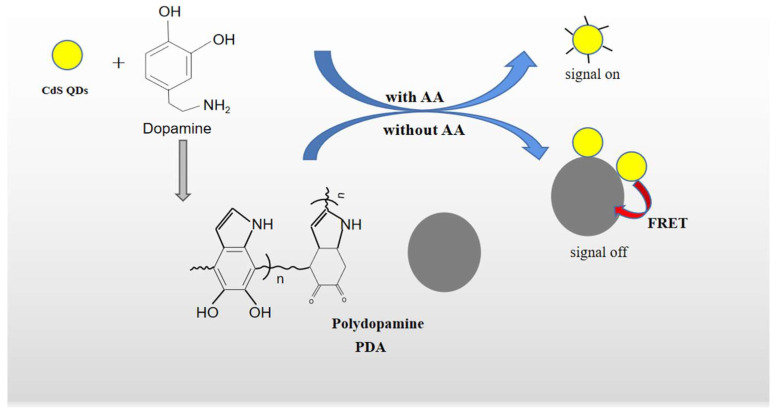
Construction principle of fluorescence signal “off” and “on” AA sensor.

Hence, we developed a fluorescence “off-on” assay for AA by means of CdS QDs coupled with PDA-based FRET (Figure 1). CdS QDs can be assembled on the surface of PDA with strong affinity via the covalent interaction between -COOH of mercaptoacetic acid (TGA) and -NH_2_ in the PDA, and then the fluorescence of CdS QDs (as a donor) was quenched by PDA (as an acceptor) to the “off” state via FRET, and the fluorescence-quenching mechanism between PDA and CdS QDs follows a dynamic, quenching mechanism that has been demonstrated. However, the oxidative polymerization of DA to produce PDA will be effectively suppressed and the quenching effect will be eliminated when AA exists. This is because AA can competitively terminate the spontaneous oxidative polymerization of dopamine by forming free radical cations through electron transfer, resulting in the fluorescence of CdS QDs’ recovery and being put in an “on” state [36,40].

## 2. Results and Discussion

### 2.1. Characterization

The following characterization was performed after centrifugation of CdS QDs with PDA and CdS QDs@PDA.

#### 2.1.1. TEM Characterization

**Figure 2:**
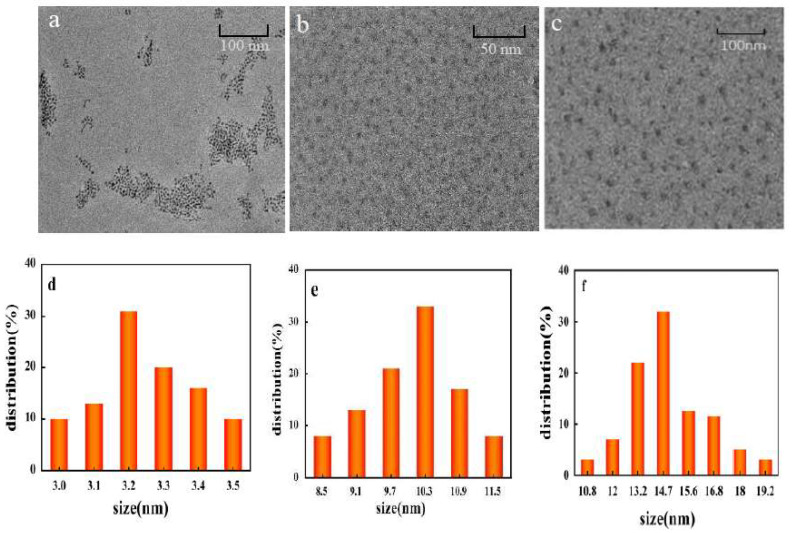
TEM images of CdS QDs (**a**), PDA (**b**) and CdS QDs@PDA (**c**). Particle size distribution of CdS QDs (**d**), PDA (**e**) and CdS QDs@PDA (**f**).

High-resolution TEM images of CdS QDs, PDA and CdS QDs@PDA are shown in Figure 2a–c. The results showed that CdS QDs, PDA and CdS QDs@PDA can be uniformly dispersed in water and the morphology were spherical or elliptical. Figure 2d–f exhibited their respective particle size distribution. It can be seen that the average particle size of CdS QDs, PDA and CdS QDs@PDA was about 3.2, 10.3 and 14.7 nm, respectively. Compared with CdS QDs and PDA, the particle size of CdS QDs@PDA was slightly larger, and the particle size distribution was more concentrated, which indirectly proved that CdS QDs was successfully assembled on the surface of PDA.

#### 2.1.2. X-ray Photoelectron Spectroscopy (XPS) Characterization of CdS QDs and CdS QDs@PDA

**Figure 3:**
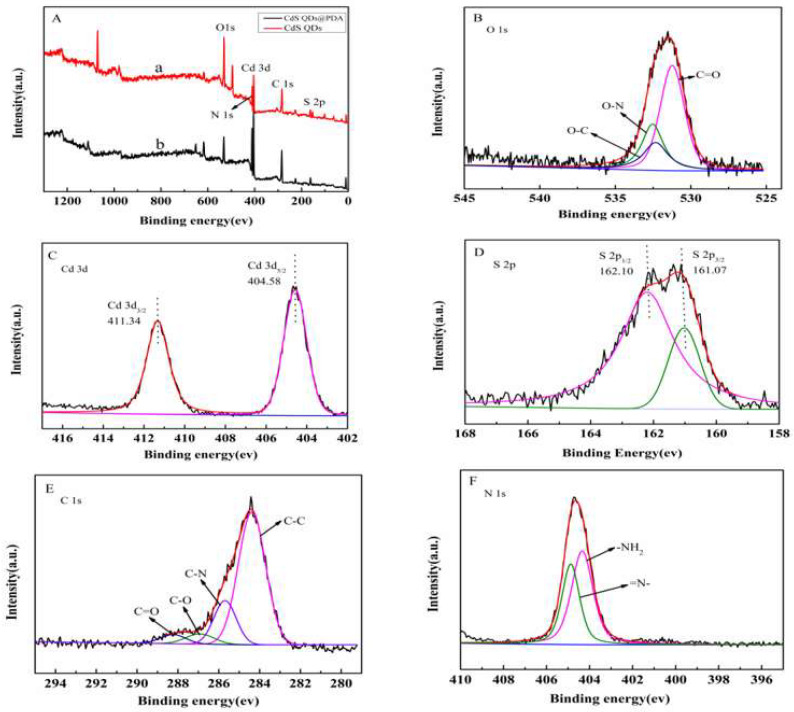
XPS spectrum (**A**) of CdS QDs (a) and CdS QDs@PDA (b). High resolution XPS spectra of O 1s (**B**), Cd 3d (**C**), S 2p (**D**), C 1s (**E**) and N 1s (**F**).

Figure 3A showed XPS images of CdS QDs (a), CdS QDs@PDA (b) and analyzed their surface’s chemical state. The peaks around 405, 160, 285, 404 and 530 eV that can be clearly observed represent the characteristic peaks of Cd 3d, S 2p, C 1s, N 1s and O 1s, respectively. The core-level XPS spectrum of O 1s (Figure 3B) exhibited three characteristic peaks at 532.38, 531.22 and 532.38 eV, which could be assigned to C-O, C=O and O-N bonds [41,42]. Figure 3C exhibited the high-resolution Cd 3d spectrum. The peaks at 411.34 and 404.58 eV are Cd 3d_3/2_ and Cd 3d_5/2_ diffraction peaks [43,44]. Figure 3D displayed the high-resolution S 2p spectrum which peaks at 162.10 eV and 161.07 eV, and can be ascribed to S 2p_1/2_ and S 2p_3/2_ [45,46], which belongs to C=S and Cd-S bond. High resolution C 1s spectrum (Figure 3E) could be deconvoluted into four characteristic peaks at 284.40, 285.80, 286.51 and 287.21 eV, which attributed to C-C, C-N, C-O and C=O bonds. The N 1s peaks (Figure 3F) at 404.35 and 404.89 eV could be associated with =N- and -NH_2_ groups [42]. The results from XPS characterizations confirmed that CdS QDs have been assembled on PDA surface.

#### 2.1.3. FTIR Characterization of DA and CdS QDs@PDA

**Figure 4:**
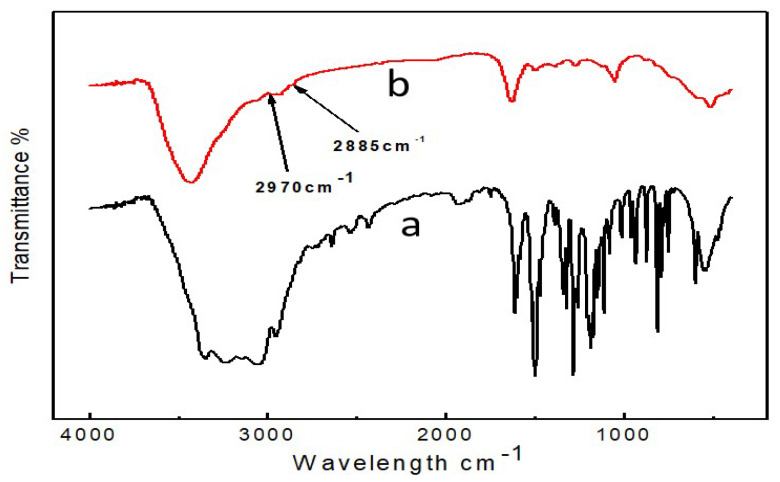
FTIR spectra of DA (a) and CdS QDs@PDA (b).

As illustrated in Figure 4, the strong, stretching, vibration-absorption peaks of N-H and O-H bond were a wide peak of 3500–3100 cm^−1^, and the weak stretching vibration absorption peaks of C-H bond appeared at 2970 and 2885 cm^−1^ [47,48]. Compared with curve a, the absorption spectrum changed dramatically between 1700 and 500 cm^−1^. The characteristic absorption peak of the DA molecule almost completely disappeared due to the generation of PDA and CdS QDs@PDA [49].

#### 2.1.4. UV-Vis and Fluorescence Lifetime Characterization

**Figure 5:**
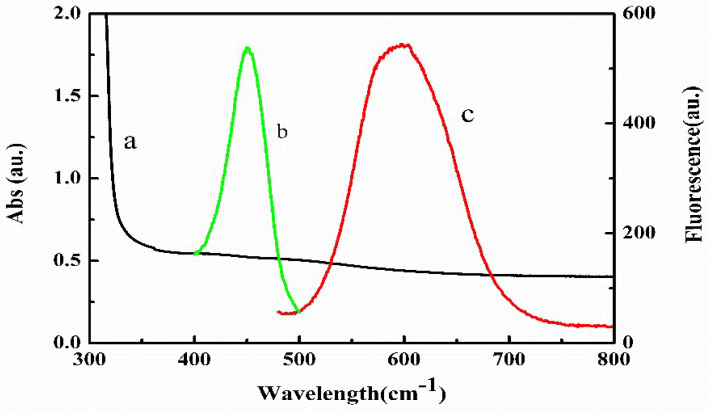
Absorption spectrum of PDA (a), excitation (b) and emission (c) spectra of CdS QDs.

**Figure 6:**
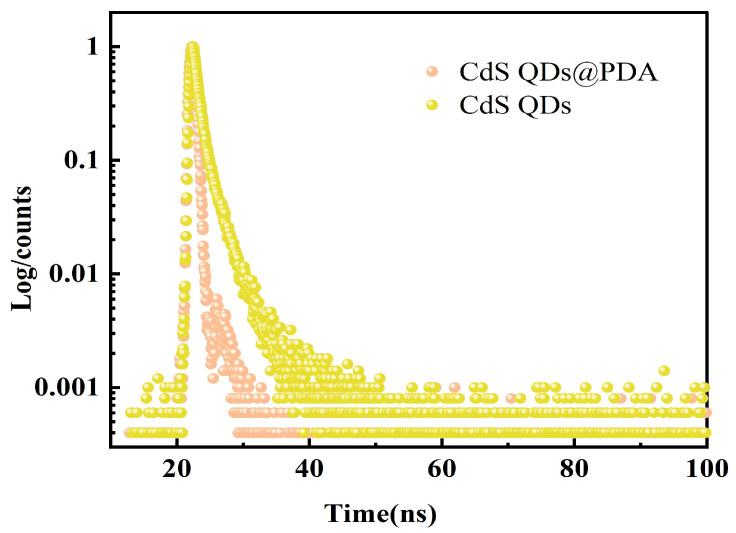
The luminescence time decay resolution curves of CdS QDs in the absence and presence of PDA (5 mmol/L DA).

Figure 5 displayed the absorption spectrum of PDA and the excitation and emission spectra of CdS QDs. It can be seen that the absorption spectrum of PDA was continuous between 300 and 800 nm, while the maximum excitation and emission wavelength of CdS QDs was near 445 and 593 nm. It was clear that the emission spectrum of the CdS QDs completely overlapped with the absorption spectrum of the PDA. The fluorescence quantum yield of CdS QDs was calculated to be about 2.4% using quinine sulfate (0.1 mol/L H_2_SO_4_) as the standard material. Additionally, the fluorescence lifetime of CdS QDs was significantly shorter in the presence of PDA than in its absence (Figure 6), indirectly proving the occurrence of FRET, which leads to fluorescence quenching of CdS QDs [50]. Therefore, the fluorescence quenching of CdS QDs by PDA followed the dynamic quenching mechanism.

### 2.2. Optimum Proposal

#### 2.2.1. Study on Fluorescence Quenching and Recovery of CdS QDs

**Figure 7:**
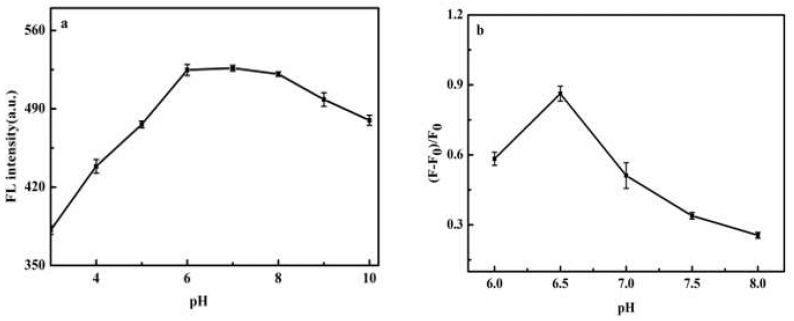
Effect of pH on fluorescence intensity of CdS QDs (**a**) and fluorescence quenching rate of CdS QDs by PDA (**b**).

Figure 7a shows the effect of pH on the fluorescence emission of CdS QDs. The fluorescence intensity of CdS QDs was relatively stable when the pH was 6.0–8.0. When pH was below 6.0, the fluorescence intensity decreases, probably because the acid (H^+^) could etch the surface S^2−^ to form HS^−^/H_2_S, which would generate surface defects [51]. When pH exceeded 8.0, it may be due to the difficulty of CdS QDs forming, or more defective CdS QDs forming at higher pH environments [52]. Figure 7b displayed the variation of recovery rate with pH. It can be seen that (F − F_0_)/F_0_ increases and then decreases rapidly with increasing pH (F and F_0_ refer to the fluorescence intensities of CdS QDs in the presence and absence of AA, respectively), with the greatest (F − F_0_)/F_0_ at pH 6.5. This pH dependence may be related to the pKa value of 4.10 for AA. At pH > 4.10, the ascorbic acid anion is the dominant species, and this anion readily precipitates stable radicals from resonance with a high reducing capacity, so that (F − F_0_)/F_0_ increases gradually with increasing pH in the range of 6.0–6.5. However, in neutral- and weakly basic solutions, AA is unstable and easily oxidized by dissolved oxygen. Thus, (F − F_0_)/F_0_ decreases rapidly with increasing pH in the range of 6.5–8.0 [53]. Therefore, 0.1 mol/L PBS at pH 6.5 was chosen throughout the reaction.

#### 2.2.2. Effect of DA Concentration and Reaction Time on Fluorescence Quenching of CdS QDs

**Figure 8:**
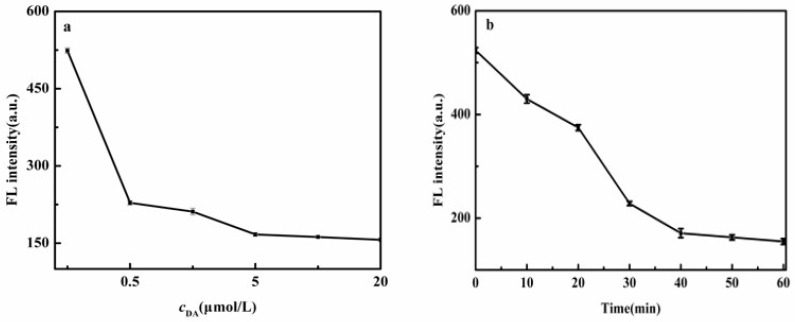
Effect of DA concentration (**a**) and quenching reaction time (**b**) on fluorescence intensity of CdS QDs.

Figure 8 exhibited that the fluorescence intensity of CdS QDs gradually decreased with the increase in DA concentration (a) and quenching reaction time (b). This was the enhancement of FRET due to an increase in the generated PDA with the increase in DA concentration [54]. When DA concentration was 5.0 mmol/L, and the reaction time reached 40 min, the fluorescence quenching rate tended to balance. Hence, for DA (5 mmol/L), the quenching reaction time of 40 min was selected for subsequent experiments.

### 2.3. Analysis Characteristics of Sensors

#### 2.3.1. Linear Range and Detection Limits

**Figure 9:**
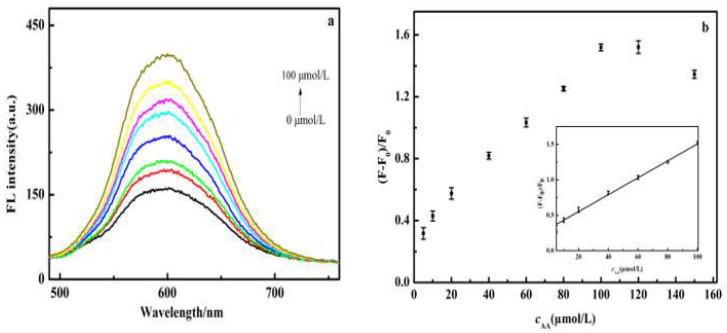
(**a**) Fluorescence spectra of sensor in the presence of different concentrations of AA. (**b**) Linear calibration curve for detection of AA.

The detection performance of CdS QDs@PDA for AA was further studied under the optimal conditions. As shown in Figure 9a, the fluorescence intensity of CdS QDs recovered gradually with the increase of AA concentration and the relationship between the fluorescence recovery rate of CdS QDs and AA concentration satisfied the following equation:

$$\frac{F-F_{0}}{F_{0}}=kc_{AA}+b\;(k\;\mathrm{is}\;\mathrm{the}\;\mathrm{quenching}\;\mathrm{constant})$$

As shown in Figure 9b, a good linear relationship was demonstrated with the concentration of AA in the range of 5.0 to 100.0 μmol/L. The linear equation was $y=0.0119c_{AA}+0.3113$ (R^2^ = 0.9959) and the limit of detection (LOD) was 1.16 μmol/L (S/N = 3, *n* = 10).

#### 2.3.2. Selectivity

**Figure 10:**
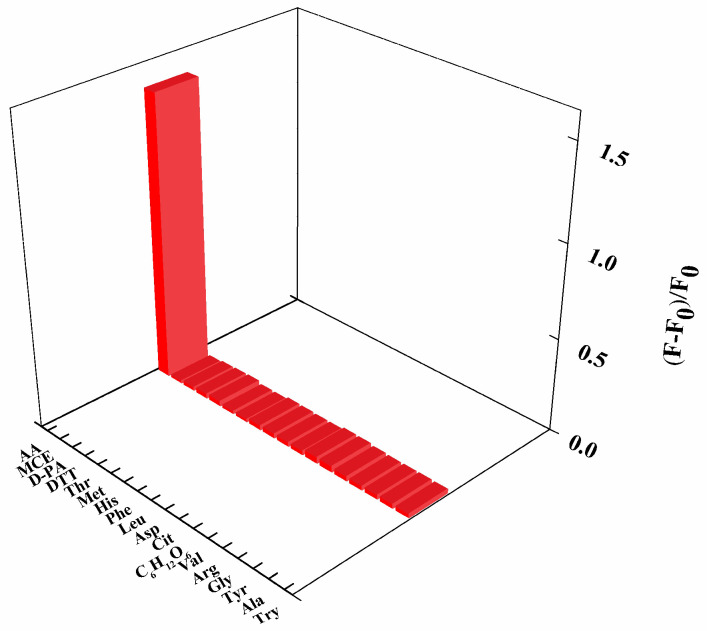
Selectivity of the proposed CdS QDs based fluorescent sensor for AA detection.

To investigate the selectivity of the fluorescent sensor for AA, seventeen kinds of representative substances were selected for the interference test at a constant concentration of 100 μmol/L (the same concentration as AA), including amino acid (Try, Ala, Tyr, Gly, Arg, Val, Cit, Asp, Leu, Phe, His, Met, Thr), bio-sulfhydryl small molecules (MCE, D-PA, DTT) and glucose, and were added independently into the solution of CdS QDs. The results are illustrated in Figure 10. A strong fluorescence quenching was observed against AA but not on other individual interfering substances, demonstrating that the sensor has good immunity to interference when interfering substances co-exist. Thus, the sensor we designed had a better selectivity for AA. 

#### 2.3.3. Actual Sample Analysis and Recovery

As can be seen from Table 1, the determination of AA in vitamin C tablets by this method was basically consistent with the labeled amount (0.1 g). The recoveries were observed to be 98.01–100.7%.

## 3. Experiment

### 3.1. Material and Reagents

Alanine (Ala), Glycine (Gly), Arginine (Arg), Tryptophane (Try), Valine (Val), Tyrosine (Tyr), Citrulline (Cit), Aspartic acid (Asp), Leucine (Leu), Phenylalanine (Phe), Threonine (Thr), Histidine (His), Methionine (Met), Dithiothreitol (DTT), Dopamine (DA), D-penicillamine (D-PA), Ascorbic acid (AA), Chromic chloride (CdCl_2_) and Sodium sulfide (Na_2_S) were purchased from Aladdin Co., Ltd., Shanghai, China; 2-mercaptoethanol (MCE), Glucose (C_6_H_12_O_6_) and Sodium hydroxide (NaOH) were obtained from Sinopharm Chemical Reagent Co., Ltd., Zhejiang, China; Mercaptoacetic acid (TGA) was supplied by Sigma-Aldrich Co., Ltd., St. Louis, MO, USA; Tris hydrochloride (Tris-HCl) was acquired from Adamas reagent Co., Ltd., Shanghai, China; vitamin tablets were purchased from Huazhong Pharmaceutical Co., Ltd., Xiangyang, China; the experimental water was ultrapure water.

### 3.2. Apparatus

The UV-vis absorption spectra were carried out using Lambda 950 spectrophotometer (PerkinElmer Inc., Waltham, MA, USA). The surface morphology of the prepared materials was characterized via S-4800 Scanning Electron Microscope (JEOL, Zaventem, Belgium). The X-ray photoelectron spectrometer was conducted on ESCALAB 250Xi X-ray photoelectron spectroscopy (Reonicolai Inc., New York, NY, USA). The FT-IR spectra was recorded on Thermo Nexus 670 infrared spectrometer (Reonicolai Inc., New York, NY, USA). Fluorescence (FL) spectra were performed on RF-6000 Fluorescence Spectrometer (Shimadzu, Kyoto, Japan). Fluorescence lifetime spectra are determined by FLS 980 Fluorescence Spectrometer (Tenmei, Edinburgh, UK). Plus-E3-20TH Ultrapure Water Device (Nieolet, MN, USA). PHS-3C pH meter (Shanghai Yidian Scientific Instrument Co., Ltd., Shanghai, China), 1810D Electronic Analytical Balance (Saidoris, Baden-Württemberg, Germany).

### 3.3. Synthesis of CdS QDs

CdS QDs was synthesized as previously reported [55]. In total, 50 mL 0.01 mol/L CdCl_2_ and 250 µL TGA were added to a 150 mL tri-neck flask. The pH was set to 11.0 using NaOH solution (1 mol/L), and oxygen was removed through N_2_ for 30 min. Then, 5.0 mL 0.1 mol/L Na_2_S solution was added before restarting the flow at 100 °C for 4 h under the protection of nitrogen. CdS QDs was obtained and kept at 4 °C for further use.

### 3.4. Optimization of Fluorescence Quenching Conditions

In total, 200 μL of CdS QDs were added to 2 mL centrifuge tubes, diluted and fixed with a pH of 3.0, 4.0, 5.0, 6.0, 7.0, 8.0, 9.0 and 10.0 buffer solution. Fluorescence was measured to determine the optimal emission pH range of CdS QDs and select the buffer system. Different concentrations of DA were then added simultaneously to select the optimum quenching pH, DA concentration and sonication reaction time.

### 3.5. Determination of Ascorbic Acid

CdS QDs (200 μL) were mixed with a series of aqueous solution of AA at different concentrations. DA (5 mmol/L) was added to the mixture and diluted with a phosphate buffer (pH = 6.5, 0.1 mol/L) to 2 mL (the final concentration of AA was 0.00, 5.00, 10.0, 20.0, 40.0, 60.0, 80.0, 100.0, 120.0 and 150.0 µmol/L). The fluorescence intensity was determined with the excitation wavelength of 445 nm after 40 min of ultrasonic reaction.

### 3.6. Actual Sample Handling

Vc tablets were measured using the same method as the actual samples. The mass of each tablet was obtained by accurately weighing 100 pieces of Vc and ground into powder. Vc powder of a certain quality was accurately weighed, dissolved in water to a certain volume and filtered. The filtrate was diluted step by step to the appropriate concentration for determination.

## 4. Conclusions

An “off” and “on” fluorescence sensing platform was constructed for the sensitive detection of AA based on the FRET between CdS QDs and PDA. The sensitive and rapid detection of AA in Vc tablets were successfully achieved. It can be foreseen that this technique can be extended to the determination of AA in other samples. At the same time, different signal sources can be used to construct a new sensor with PDA to realize the detection of various reductants.

## Figures and Tables

**Table 1:** The content and recovery of AA in vitamin C tablets samples.

Sample	Labeled (g/Piece)	Detected/μmol·L^−1^ (x±SD,n=3)	Added /μmol/L	Found/μmol/L (x±SD,n=3)	Recovery/% (x±SD,n=3)
Vc tables	0.1	7.28 ± 0.61	7.50	14.63 ± 0.11	98.01 ± 1.47
		20.23 ± 0.80	20.00	40.19 ± 0.81	100.7 ± 1.55
		40.88 ± 1.68	40.00	80.46 ± 1.05	99.01 ± 2.60

## Data Availability

Not applicable.

## Sample Availability

Samples of the compounds in this article are not available from the author.
